# Consistency and interpretation of changes in millimeter-scale cortical intrinsic curvature across three independent datasets in schizophrenia^☆^

**DOI:** 10.1016/j.neuroimage.2012.06.034

**Published:** 2012-10-15

**Authors:** Lisa Ronan, Natalie L. Voets, Morgan Hough, Clare Mackay, Neil Roberts, John Suckling, Edward Bullmore, Anthony James, Paul C. Fletcher

**Affiliations:** a Brain Mapping Unit, Department of Psychiatry, University of Cambridge, Cambridge, UK; b FMRIB Centre, Nuffield Department of Clinical Neurosciences, University of Oxford, Oxford, UK; c Clinical Research Imaging Centre, Queen's Medical Research Institute, University of Edinburgh, Edinburgh, UK; d Highfield Adolescent Unit, Warneford Hospital, Oxford, UK

**Keywords:** Intrinsic curvature, Gyrification, Cerebral cortex, Schizophrenia, Connectivity

## Abstract

Several studies have sought to test the neurodevelopmental hypothesis of schizophrenia through analysis of cortical gyrification. However, to date, results have been inconsistent. A possible reason for this is that gyrification measures at the centimeter scale may be insensitive to subtle morphological changes at smaller scales. The lack of consistency in such studies may impede further interpretation of cortical morphology as an aid to understanding the etiology of schizophrenia.

In this study we developed a new approach, examining whether millimeter-scale measures of cortical curvature are sensitive to changes in fundamental geometric properties of the cortical surface in schizophrenia. We determined and compared millimeter-scale and centimeter-scale curvature in three separate case–control studies; specifically two adult groups and one adolescent group. The datasets were of different sizes, with different ages and gender-spreads. The results clearly show that millimeter-scale intrinsic curvature measures were more robust and consistent in identifying reduced gyrification in patients across all three datasets.

To further interpret this finding we quantified the ratio of expansion in the upper and lower cortical layers. The results suggest that reduced gyrification in schizophrenia is driven by a reduction in the expansion of upper cortical layers. This may plausibly be related to a reduction in short-range connectivity.

## Highlights

► Studies of cortical gyrification in schizophrenia have to date been equivocal. ► Millimeter-scale measures of gyrification are potentially more sensitive and robust. ► Intrinsic curvature can be used to quantify gyrification at the millimeter scale. ► mm-scale gyrification is consistently reduced across multiple schizophrenic groups. ► Reduced gyrification predominantly reflects changes in upper cortical layers.

## Introduction

A major challenge in schizophrenia research is to identify and characterize a core pathophysiology that could account for the wide array of symptoms. Clearly, the deficit must be diffuse and pervasive (White and Hilgetag, 2011), a fact that has been reflected in an emphasis on explanatory models that appeal to disruptions in neural connectivity (Harrison, 1999). Such ideas have been embedded in more specific theories such as cognitive dysmetria (Andreasen and Paradiso, 1998; Andreasen et al., 1999), cerebral asymmetry (Crow, 2008), and the reduced neuropil hypothesis (Selemon and Goldman-Rakic, 1999), each implicating aberrant connectivity as the structural substrate for functional abnormalities. These theories have been supported by a wealth of independent evidence ranging from abnormal functional connectivity (Honey et al., 2005; Micheloyannis et al., 2006), loss of asymmetry in minicolumn separation and cortical volumes (Bilder et al., 1994; Turetsky et al., 1995), and reduced inter-neuronal spacing (Buxhoeveden et al., 2000). However, a striking aspect of the findings from brain imaging studies of schizophrenia is the heterogeneity of observations, with many studies failing to replicate findings or even producing conflicting results (Chua and McKenna, 1995; Shenton et al., 2001).

Although the structural evidence is inconclusive, there is a general consensus that schizophrenia is, at least partly, a disorder of neurodevelopment (Rapoport et al., 2005; Weinberger, 1987). Consistent with this view is a lack of gliosis (Roberts et al., 1987), the presence of structural abnormalities at an early stage in first-episode patients, and the presence of behavioral abnormalities prior to the onset of symptoms (Khandaker et al., 2011).

In an attempt to account for both abnormal neurodevelopment and abnormal connectivity, some studies have sought to identify changes in cortical gyrification (for a review see White and Hilgetag, 2011; Mangin et al., 2010). Gyrification is the process of tangential surface expansion by which an increasingly large surface area is fit into a relatively small volume (Pillay and Manger, 2007). With expansion, the initially smooth surface begins to buckle. Although the factors that govern this buckling are still under examination, most theorists concur in relating it to underlying cortical connectivity (Richman et al., 1975; Van Essen, 1997). The degree of buckling – gyrification – is generally quantified as the relative increase in surface area of the cortex compared to a reference surface such as a convex hull (Zilles et al., 1988): a larger ratio implying a greater degree of gyrification. Gyrification is temporally non-linear (Armstrong et al., 1995), and is influenced by intra-uterine factors affecting cortical formation and modulating subsequent neurobehavioral development (Dubois et al., 2008).

Given that gyrification is shaped by development and connectivity, it ought to be a useful measure in schizophrenia. Disappointingly, however, case–control studies have produced equivocal results, with some reporting increase (Harris et al., 2007; Narr et al., 2004; Vogeley et al., 2001), others decreases (Cachia et al., 2008; Penttila et al., 2008; McIntosh et al., 2009), and some no change (Highley, 2002). Clinical heterogeneity, methodological differences (including the various use of two or three-dimensional parameters), cohort size and different selection of regions of interest have all been suggested as reasons for these discrepancies. However a further reason for this inconsistency may lie in the scale at which these measures are made. Typically, gyrification is measured at the centimeter scale, but this may be sub-optimally gross. Sulci and gyri are themselves the aggregate of biological components and forces such as neuronal proliferation and cortical expansion that occur at scales many orders smaller. As such, subtle differences in the factors that contribute to cortical morphology may be obfuscated when measured at larger scales. Central to the present paper, we propose an alternative method that, by quantifying differences in cortical morphology at a millimeter-scale across the cortex, may enable a more sensitive and reliable identification of differences in morphology and, hence, distinguish more consistently between schizophrenic patients and healthy controls.

**Fig. 1:**
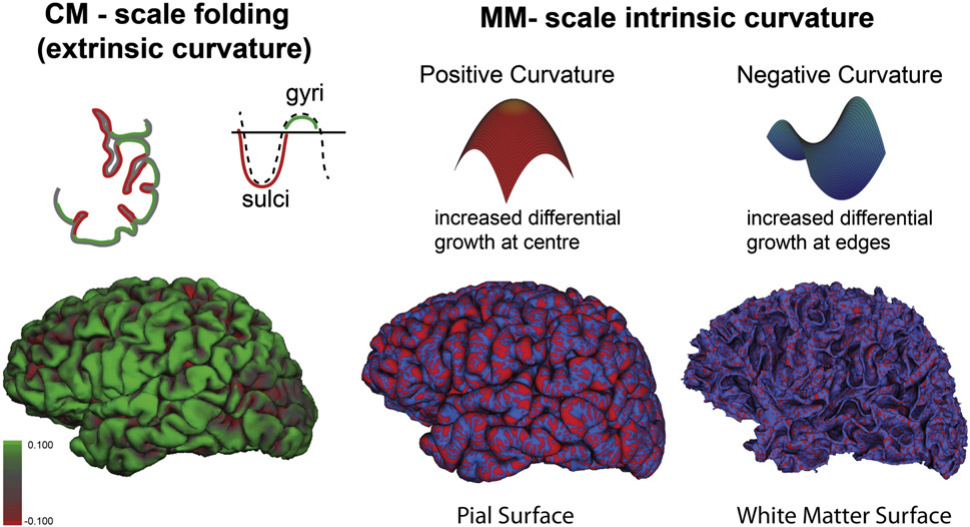
The expansion of the cortex causes it to fold, producing gyri (green) and sulci (red) at the centimeter scale (illustrated in terms of smoothed mean curvature). However at the millimeter scale, this expansion may give rise to intrinsically curved shapes. The spatial frequency of morphometric patterns varies depending on the scale measurements made; at the centimeter-scale, the cortical folding of the cortex has a low frequency, whereas at the millimeter-scale, the pattern of intrinsic curvature (positive intrinsic curvature red; negative intrinsic curvature blue) is much higher and does not follow the pattern of sulcal/gyral features. Under the hypothesis that intrinsic curvature is a morphological parameter of differential surface expansion, as the degree of differential expansion increases, there is a corresponding increase in the degree of intrinsic curvature and hence the ratio of surface area to a flat plane. Because of this, intrinsic curvature may be used as a marker of cortical gyrification analogous to the more ubiquitous centimeter-scale folding-based measures.

A crucial, if not often explored, aspect of cortical morphology is the distinction between the extrinsic and intrinsic surface properties (Griffin, 1994). During development, the expansion of the cortical surface is associated with the characteristic folding of the cortex, an extrinsic property in the sense that the folding is not a function of the surface itself, but rather reflects how it is embedded in space. As well as folding, developmental expansion may also give rise to another type of curvature, namely intrinsic curvature (Ronan et al., 2011) (see Fig. 1). This is so called because it is intrinsic to the shape of the surface, i.e. unlike folding it cannot be removed without deforming or tearing the surface. Intrinsic curvature may be conceptualized as a product of differential expansion. It may be visualized thus: if an area expands at a uniform rate in all directions the surface will remain flat, however if the center grows faster than the edges, it will induce a positive curvature with a shape similar to a hemisphere, if the edges grow more quickly than the center the result will be a hyperbolic surface (saddle-shaped) (Fig. 1). The greater the rate of differential growth, the greater the degree of induced curvature in the surface, and hence the greater the increase in surface area relative to a flat plane. Thus intrinsic curvature may be thought of as capturing the surfeit or deficit of surface area relative to a flat plane. In this context, measures of the degree of intrinsic curvature are analogous to other more commonly applied measures of gyrification that quantify the ratio of one surface (the folded cortex) to another (smooth reference surface). Crucially, using high-resolution cortical surface reconstruction techniques, we can now quantify the degree of intrinsic curvature at a millimeter-scale (Ronan et al., 2011), thus offering a potential increase in the power to detect subtle differences in cortical morphology in schizophrenia.

In summary, the tangential expansion of the cortex causes the surface to fold at a centimeter-scale (producing gyri and sulci), while simultaneously producing intrinsic curvature shapes (positive and negative curvatures) at the millimeter-scale. By comparing each morphological shape (folding and intrinsic curvature) to a reference surface we can generate measures of cortical gyrification at two distinct scales. We propose that intrinsic curvature at the millimeter-scale may be an intermediary measure between neuronal processes at one end and larger-scale gyri and sulci at the other. It thus may provide a more sensitive method for distinguishing subtle differences in cortical morphology between groups.

Developing a more sensitive measure of gyrification is useful, especially if it can help to resolve ambiguities that currently exist in the schizophrenia literature. However it is important, too, that such observations may be used to establish a deeper understanding of the etiology of schizophrenia. One method of exploring this is to contrast the relative expansion of cortical layers. Alterations in neuronal populations between cortical layers have been shown to relate to alterations in gyrification (Richman et al., 1975), and, furthermore, although all layers in the cortex contribute to connectivity, the upper layers have on average more short-range intra-cortical connectivity, whereas lower layers have more long-range, inter-cortical connectivity (Valverde et al., 1985). Thus, as proposed previously (Armstrong et al., 1991), differences in the ratio of upper to lower layer expansion can be related to altered gyrification patterns (Richman et al., 1975), as well as used to make inferences about the relative proportions of short-to-long range cortical connectivity. Hence, under the hypothesis that intrinsic curvature is a morphological marker of differential surface expansion, it may be used to lend further insights in to the connectivity of the cortex.

In this experiment we characterized cortical gyrification at the (standard) centimeter scale, and at the millimeter-scale (using intrinsic curvature). Under the hypothesis that both large scale folding and small-scale intrinsic curvature are functions of surface expansion, we hypothesized that the standard gyrification measures would show a relationship with intrinsic curvature, but that the latter would more consistently identify group differences. In order to test this hypothesis we assessed both scales of gyrification in three independent case–control datasets (two adult groups and one adolescent group) and compared the results. The results of our analysis were in agreement with our hypothesis that millimeter-scale intrinsic curvature was more sensitive to case–control differences in each group. To interpret our gyrification findings in the context of connectivity, we further characterized the ratio of intrinsic curvature (corrected for surface area) between the outer (pial) and inner (white matter) surface.

It should be noted that our comparison of the performance of traditional gyrification and the intrinsic curvature measures entails some ambiguity given that these measures operate at different scales as well as measuring different characteristics. Therefore, in a further experiment we sought to determine whether the increased sensitivity of intrinsic curvature was due to the scale at which it was measured or its geometric nature (i.e. an intrinsic as opposed to an extrinsic surface parameter). To this end, in a series of analyses, we contrasted intrinsic curvature between groups at increasing scales approaching the centimeter-domain. We additionally quantified case–control differences in mean curvature (an extrinsic surface measure which reflects surface folding) at the millimeter scale and contrasted this to our millimeter-scale intrinsic curvature results.

## Methods

### Subjects and MR acquisition parameters

Three different datasets were analyzed; adolescent-onset schizophrenia (AOS, with age at onset of symptoms < 18 years old) in a longitudinal dataset with two time points (baseline, follow-up), and two adult-onset groups (Group 1, Group 2). Data for adolescents and adults (Group 1) was collected with the guidance of the Oxford and Berkshire Psychiatric Research Ethics Committees, UK. Data for adults (Group 2) was collected under the guidance of the NHS Trust Local Research Ethics Committee, Cambridge, UK. Written informed consent was obtained from all participants (and their parents if under the age of 16).

#### Adolescents

Seventeen AOS patients (9 males) (16.1 ± 1.1 years) and fifteen age-matched normal control subjects (10 males) (15.7 ± 1.3 years) were recruited over a period of 2 years from the Oxford regional adolescent unit and surrounding units. All were diagnosed as having DSM-IV schizophrenia using the Kiddie Schedule of Affective Disorders and Schizophrenia (Kaufman et al., 1997). The mean interval between scans was approximately 2 years for each group, and did not differ between groups. Age at follow-up was 18.2 (± 1.4 years) for patients, and 18 (± 1.6 years) for controls.

Structural MRI data were acquired using a 1.5 T Sonata MR imager (Siemens, Erlangen, Germany) with a standard quadrature head coil and maximum 40 mT m^− 1^ gradient capability at the Oxford Centre for Clinical Magnetic Resonance Research (OCMR). Whole brain T1-weighted images were acquired with a FLASH sequence using the following parameters: coronal orientation, image matrix = 256 × 256, with 1 × 1 mm^2^ in-plane resolution, 208 slices of slice thickness 1 mm, TE = 5.6 ms, TR = 12 ms, and flip angle α = 19°.

#### Adult: Group 1

Forty-six patients (36 males) (33.2 ± 9 years) were recruited by collaborating psychiatrists from Oxfordshire and Berkshire Mental Healthcare Trusts. Diagnosis was confirmed using the Structural Clinical Interview for DSM-IV Disorders (First et al., 1997). Forty-four controls (32 males) were also recruited (30.4 ± 8 years). There were no statistically significant differences in age between patients and controls. MR acquisition parameters were as above.

#### Adult: Group 2

Thirteen patients (8 males) (24.8 ± 4.7 years) were recruited from within the Cambridgeshire and Peterborough Mental Health Partnership NHS Trust, with a first episode of psychosis that satisfied the DSM-IV criteria for schizophrenia. Thirteen controls (9 males) (26.5 ± 9 years) were also recruited. There were no statistically significant differences in age between patients and controls.

Structural MRI data were acquired on a GE Signa HDxt system (General Electric, Milwaukee WI, USA) operating at 3 T at the Department of Radiology, University of Cambridge. Whole-brain T1-weighted images were acquired with an inversion recovery prepped, fast 3D gradient-recalled echo sequence with the following parameters: parallel to the ac–pc line, image matrix = 512 × 512, with 0.4688 × 0.4688 mm^2^ acquired in-plane resolution, and 120 slices of slice thickness 1.1 mm, TE = 3880 ms, TR = 9060 ms, and flip angle α = 20°.

### Cortical reconstruction and analysis

Cortical reconstructions were generated using the software *FreeSurfer* (Dale et al., 1999; Fischl et al., 1999; Fischl and Dale, 2000). The *FreeSurfer* program was specifically developed for cortical reconstruction. In brief, raw image data voxels are sub-sampled to voxels of side 1 mm^3^. After that the data is normalized for intensity, RF-bias field inhomogeneities are modeled and removed, followed by skull-stripping. The cerebral white matter is subsequently identified after which the hemispheres are separated, tessellated and deformed to produce an accurate and smooth representation of the gray–white interface. These surface reconstruction processes are conducted in native space. In case of inaccuracies, the reconstructions may be edited by hand. These edits are made on two-dimensional slices though the reconstruction and hence may be considered to be effectively unbiased with respect to the morphological parameters which are three-dimensional. The *FreeSurfer* program has been demonstrated to be robust to differing scanner types and field strengths (Han et al., 2006).

#### Measuring gyrification

Gyrification was assessed in two different ways, first at the centimeter-scale using a three-dimensional measure called the local gyrification index (*lGI*) (Schaer et al., 2008), and then at the millimeter-scale by measuring intrinsic curvature of the cortex (Pienaar et al., 2008; Ronan et al., 2011).

##### *lGI*

The *lGI* is a ratio of the total cortical surface area to a reference surface, with higher indices implying a greater degree of gyrification. In brief, the *lGI* is calculated per vertex as the ratio of surface areas between a patch of cortical area which follows the folding of the cortex, and the area of a smooth reference surface (with radius of 25 mm). This outer surface encloses and hence defines the area of the cortical patch for each vertex. It is the ratio of these areas that generated the *lGI* per vertex, and is considered to be a centimeter-scale measure of the local folding of the cortex. Further details of these methods are available in Schaer et al. (2008).

For each subject the average *lGI* was calculated across each hemisphere.

##### Gaussian curvature

Details of Gaussian, or intrinsic curvature have been described elsewhere (Pienaar et al., 2008; Ronan et al., 2011). Just like *lGI*, intrinsic curvature is calculated at every vertex of the *FreeSurfer-*generated cortical surface reconstruction, albeit at a millimeter-scale scale. In brief, for each vertex the Gauss–Bonnet scheme (see Appendix A, Surazhsky et al., 2003) is used to generate values of the principle curvatures of the surface at that point. In two dimensions, the curvature of a line is defined as the reciprocal of the radius of curvature of an osculating circle. For a point on a surface however there are many different possible orientations in which curvature may be calculated, generating a range of curvature values. The maximum and minimum curvature values are always generated at orientations that are orthogonal. These orientations are called the principal directions, and the respective curvatures are the principal curvatures (see Ronan et al., 2011). The product of the principal curvatures is the intrinsic, or Gaussian curvature, *K*. Thus, the Gaussian curvature per vertex is generated by multiplying together the principle curvatures at that vertex. Prior to this, the principle curvatures are filtered (see Appendix A) to remove single vertex errors in the reconstruction. After filtering, the intrinsic curvature per vertex is multiplied by the area of the vertex to correct for non-uniformity of vertex areas in the reconstruction. The robustness of this process for different levels of surface decimation has previously been investigated (see Appendix A and Ronan et al., 2011).

The final result is a filtered value of positive or negative intrinsic curvature for each vertex in the reconstruction. For further analysis, the skew of the distribution of these values (rather than the average of the distribution) is calculated per hemisphere per subject (see Curvature skew section).

#### Investigating the effects of scale and intrinsic vs. mean curvature

To assess the role of scale in the sensitivity of intrinsic curvature, we quantified intrinsic curvature over several increasingly gross scales. We additionally investigated whether the nature of intrinsic curvature as a more mathematically fundamental surface parameter contributed to its sensitivity. To this end we additionally quantified mean curvature at the millimeter scale and contrasted it with intrinsic curvature results.

##### Down-sampling the cortex to test for scale-sensitivity

In order to quantify intrinsic curvature at several different scales, we mapped each subject's original pial surface reconstruction to a standard template with three different levels of resolution (decreasing resolution: 40,962, 10,242 and 2562 vertices respectively). These templates are the standard *FreeSurfer* templates which are included in the vFS5.1 release. This mapping did not deform the shape of the surface, rather it simply generated a new surface with larger and less numerous vertices. Cortical intrinsic curvature was calculated on the down-sampled surface for each of the three levels of resolution and used to assess the effect of scale on the sensitivity of intrinsic curvature to case–control differences.

##### Mean curvature

Mean curvature is a local measure of the degree of folding of a surface. For example, a surface with a gentle fold will have a more modest mean curvature than one with a sharp fold. Mathematically there is a categorical distinction between Gaussian and mean curvature. The distinction between these types of curvature is most obvious when we contrast the intrinsic curvature of a sphere with the extrinsic mean curvature of a cylinder. Whereas it is possible to remove the mean curvature of the cylinder by unfolding it to a flat plane, it is not possible to flatten the sphere without deforming the surface. In other words intrinsic curvature is an integral part of a surface, whereas the mean curvature is not.

Experimentally the principle curvatures used to generate the Gaussian curvature per vertex are also used to generate the mean curvature at the vertex, where mean curvature is the average of the principle curvatures. Once again filtered principle curvature values are used to quantify mean curvature (see Appendix A).

While the millimeter-scale Gaussian curvature of the cortex has a spatial frequency much greater than the folds of the cortex, the millimeter-scale mean curvature reflects the extrinsic folding of gyri and sulci (see Fig. 1).

### Analysis

#### Curvature skew

As discussed above, the rate of differential expansion impacts on the degree of intrinsic curvature, with greater rates producing proportionately more extreme curvature values. However the differential component of the expansion also implies that the shape of the distribution will change, with proportionately fewer extreme curvature values produced as the rate of differential growth increases (Ronan et al., 2011). Thus, the shape of the distribution will change as well as the relative position of its average. For this reason we quantified for each subject the skew of the intrinsic curvature distribution per hemisphere, rather than the average of the distribution per hemisphere (see Appendix B). Previous studies of cortical intrinsic curvature have demonstrated that the distribution of positive and negative values is heavily weighted toward zero (Pienaar et al., 2008; Ronan et al., 2011). Thus, more extreme skew indicates a distribution more heavily weighted toward zero intrinsic curvature and hence may be interpreted as indicating less differential expansion.

#### Statistics

Numerical transforms (BoxCox) were applied where appropriate prior to linear regression analysis. Because each subject had two measures (one per hemisphere) for each morphological parameter, we used repeated measures ANOVA to assess case–control differences of curvature and *lGI* within each group, with age and surface area as covariates. We also used repeated measures ANOVA to test for sex-by-status (where “status” indicates patients and controls) interactions, and hemisphere-by-status interactions within each parameter. The values of the F (Fisher's) statistic, the degrees of freedom and the corresponding p values were reported for each test.

Regression was used to test for status-by-surface (pial vs. white matter) interactions for intrinsic curvature skew and surface area. The statistical threshold for significance was set at an alpha value of 0.05, using bilateral p-values.

## Results

### Correlation of intrinsic curvature skew with *lGI* and surface area

**Fig. 2:**
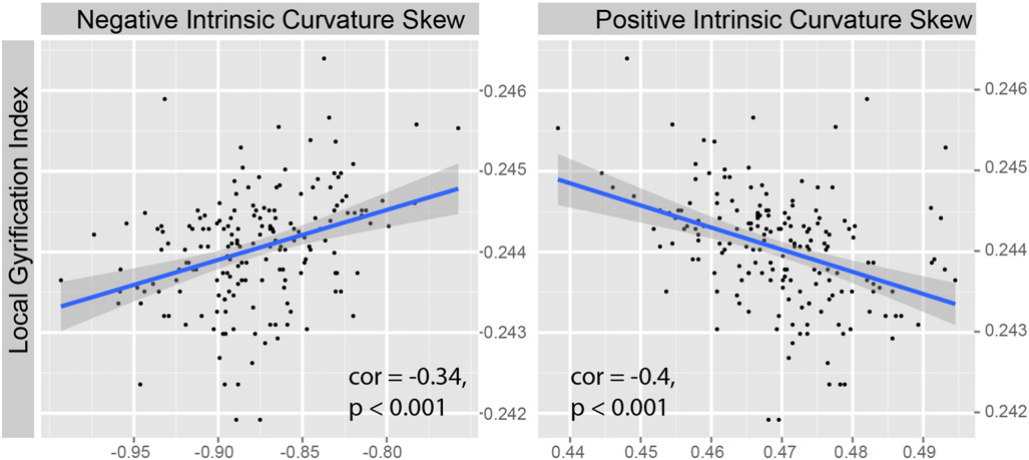
Scatter plots depicting the increase in *lGI* (larger values) with increasing degree of intrinsic curvature skew (smaller values) for positive and negative intrinsic curvatures.

When data were combined across groups, we observed a strong, positive correlation between cortical surface area and each of the gyrification parameters (negative curvature; F_1,178_ = 12.6, p = < 0.001: positive curvature; F_1,178_ = 14.1, p = < 0.001: *lGI*; F_1,178_ = 347, p = < 0.001). This is as expected, given that gyrification at both scales is a function of an increase in surface area. Unsurprisingly there were also strong correlations between *lGI* and intrinsic curvature skew (negative curvature; F_1,178_ = 24.4, p = < 0.001: positive curvature; F_1,178_ = 31.6, p = < 0.001) (see Fig. 2). This result has an implication for current gyrification theories (see Discussion).

### Intrinsic curvature and *lGI*: patients vs. controls

**Fig. 3:**
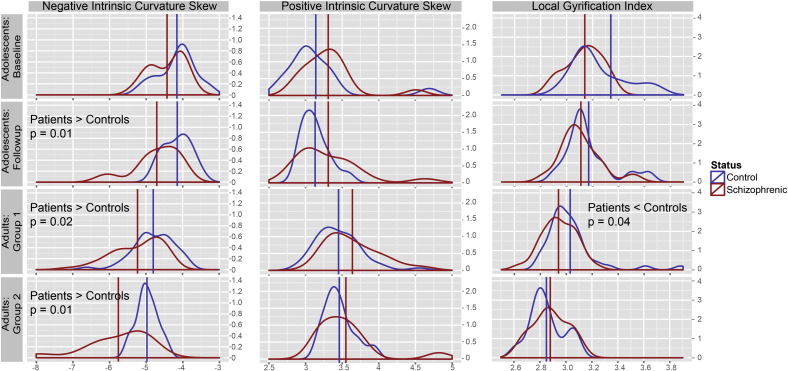
Density plots per dataset for negative and positive intrinsic curvature skew, and *lGI*. The red and blue lines indicate the average skew values for patients and controls respectively.

The results of gyrification analysis at the millimeter and centimeter scale (positive and negative curvature skews, and *lGI* respectively) are reported in Table 1 for each dataset, and illustrated in Fig. 3. For each group, patients had more extreme curvature skew values than controls. Linear analysis revealed significant differences in negative curvature in three of the four groups (adolescents follow-up F_1,23_ = 8.3, p = 0.01; adults Group 1 F_1,82_ = 5.5, p = 0.02; adults Group 2 F_1,18_ = 7.9, p = 0.01). Patients also had more extreme positive curvature skew in all datasets, but did not reach statistical significance in any group.

At the cm-scale there was also a pattern for reduced gyrification, however only the largest adult dataset (F_1,82_ = 4.2, p = 0.04) showed a statistically significant difference.

There were no significant sex-by-status interactions or hemisphere-by-status interactions for any parameter in any of the groups.

### Intrinsic curvature and cortico-cortical connectivity

In order to interpret our finding of a reduced gyrification in schizophrenia, we investigated the relative differences in intrinsic curvature of the pial and white matter surface between patients and controls. To do this we pooled all data across groups and analyzed case–control interactions at the cm-scale (using surface area), and at the mm-scale (using negative intrinsic curvature skew), with sex and age as covariates.

For the surface area analysis there was a significant difference for the pial surface (F_1,171_ = 4.7, p = 0.03) but not for the white matter surface (F_1,171_ = 1.5, p = 0.22). Linear analysis did not indicate a significant surface (pial vs. white matter) by status (patient vs. control) interaction (F_1,342_ = 0.4, p = 0.53).

**Fig. 4:**
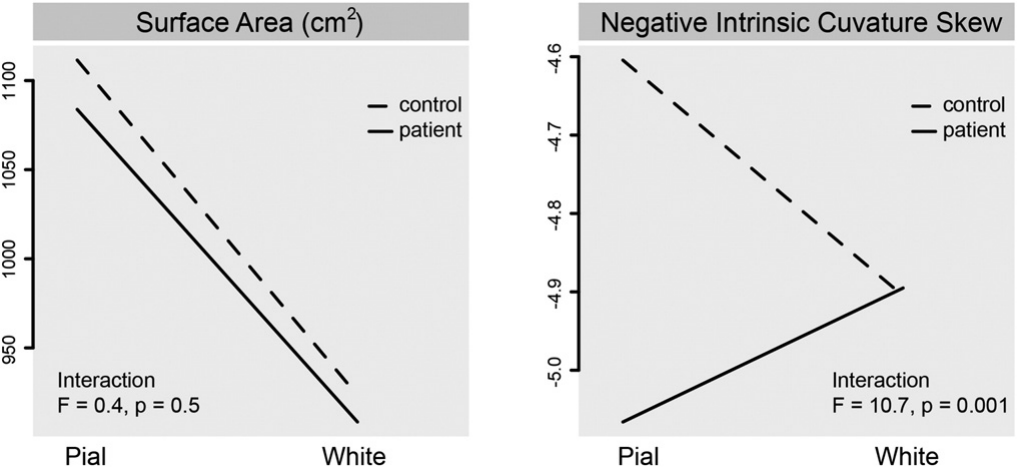
Interaction plots of surface (pial:white) and status (patient:control) for surface area and negative intrinsic curvature skew.

For negative intrinsic curvature skew, there were significant differences between patients and controls for the pial (F_1,171_ = 22.3, p < 0.001) but not for the white matter surface (F_1,171_ = 0.02, p = 0.88). Linear analysis indicated a significant surface (pial vs. white matter) by status (patient vs. control) interaction, with controls having significantly increased ratio of pial to white matter negative intrinsic curvature skew compared to patients (F_1,342_ = 10.7, p = 0.001) (see Fig. 4).

### Scaling intrinsic curvature

**Fig. 5:**
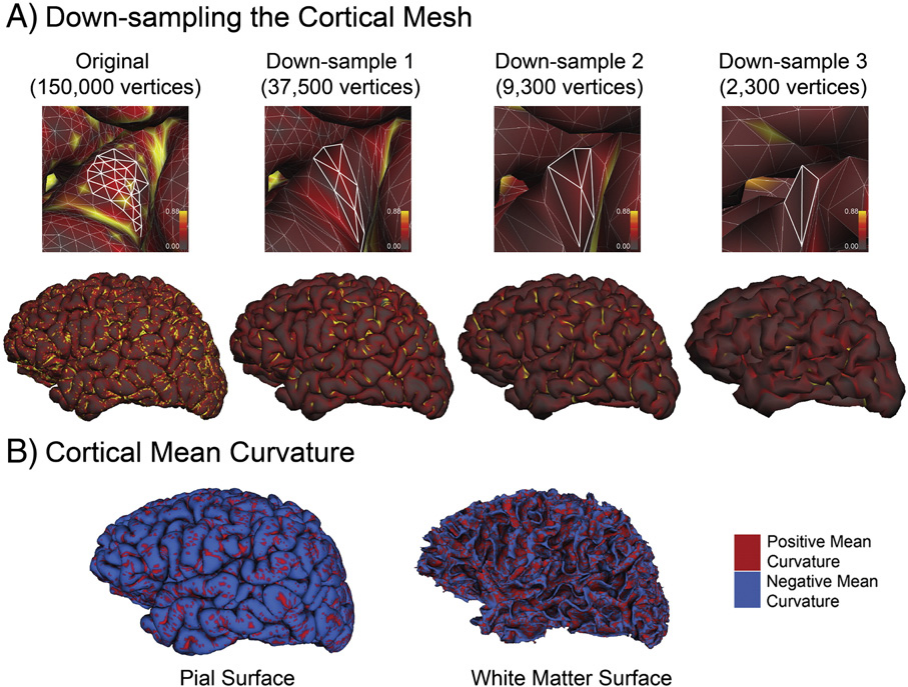
(A) The original cortical mesh of the pial surface and it's average convexity down-sampled over three scales. The vertices delineated are roughly within the same location and illustrate the degree of detail lost at each successive iteration. (B) Cortical mean curvature on the pial and white matter surface. Positive and negative mean curvatures are indicated by red and blue respectively. The spatial frequency of millimeter-scale mean curvature approximates the gyri and sulci of the cortex, and has a lower frequency than millimeter-scale intrinsic curvature (See Fig. 1).

For the original pial reconstruction the average number of vertices per hemisphere was approximately 150,000, with an average area per vertex of 0.77 mm^2^. For successive levels of down-sampling the average number of vertices and their corresponding areas were 37,500 (2.78 mm^2^), 9300 (9.9 mm^2^), and 2300 (31.9 mm^2^) (see Fig. 5).

The results of average intrinsic curvature skew for each sub-sampled reconstruction are broken down by diagnosis and dataset in Table 2, along with the results of statistical analysis of case–control differences. In summary the sensitivity to case–control differences was reduced as the intrinsic curvature was quantified at increasingly large scales.

### Mean curvature: patients vs. controls

An example of cortical mean curvature is illustrated in Fig. 5. The results of average mean curvature skew are broken down by diagnosis and dataset in Table 3, along with the results of statistical analysis of case–control differences. For each dataset there were no statistically significant differences in mean curvature between patients and controls.

## Discussion

In this study, we sought to demonstrate that millimeter-scale measures of intrinsic curvature are a more sensitive, and reliable method of quantifying cortical gyrification than the more usual centimeter-scale extrinsic measures. We further sought to use intrinsic curvature to develop deeper insights into the pathology of schizophrenia, specifically addressing what a reduced gyrification in schizophrenia might mean in terms of cortical connectivity.

The introduction of a smaller-scale measure of gyrification was motivated by the apparent inconsistencies of previous studies. Given that there is a large variability in clinical and demographic variables across gyrification studies in schizophrenia, it was important to demonstrate that smaller-scale intrinsic curvature measures were robust to these variables, and hence sensitive to any common underlying pathology. For this reason we included three independent, case–control datasets. We note that, although within-study matching was ensured, the different datasets were not matched in terms of age, gender-spread, illness duration, number of subjects or MR acquisition centers. The results of our analysis demonstrated that in all three datasets, intrinsic curvature analysis confirmed a reduction of gyrification in the schizophrenia groups. In contrast, larger-scale measures were less consistent and only identified case–control differences in the largest dataset. In summary, these results confirm our hypothesis that millimeter-scale measures of gyrification are indeed potentially more sensitive than larger-scale measures in identifying morphological abnormalities.

In the subsequent analysis, we sought to understand more fully why the millimeter-scale intrinsic curvature showed this consistent increase in sensitivity. One possibility is that it is a consequence of the smaller scale at which intrinsic curvature was quantified compared to traditional gyrification measures. Clearly, measures at increasingly smaller scales offer more detail and hence are more likely to be sensitive to case–control differences. Another possibility is that the sensitivity reflects the intrinsic nature of the Gaussian curvature as a surface parameter. The cortical surface has widespread intrinsic curvature (Griffin, 1994; Ronan et al., 2011) and it could well be that this property is key to its functional integrity, thus making intrinsic geometry an important complementary measure for characterizing alterations in schizophrenia. In order to explore these two possibilities we evaluated intrinsic curvature at different levels of resolution, as well as determined whether an extrinsic measure (mean curvature) became sensitive to group differences when it was carried out at the millimeter scale. The results of these analyses demonstrate that at the millimeter scale, intrinsic but not extrinsic curvature measures were sensitive to case–control differences. Moreover, with increasing scale, the intrinsic curvature measure showed reduced sensitivity. Taken together these results suggest that it is the intrinsic nature of Gaussian curvature in addition to its scale that is key to the reported sensitivity. In short, we have demonstrated that it is not only the scale of the measure but also the geometric nature of the measure that is important in generating a reliable and consistent biomarker of cortical morphology and its alteration in schizophrenia.

Intrinsic curvature at the millimeter-scale has a high spatial frequency, reflecting the small scale at which it is measured. Because of this high spatial frequency, is it not feasible to use this measure for more standard per-vertex measures across the cortical surface, however other parameters based on the intrinsic geometry of the cortex (Griffin, 1994) might be adapted for this purpose in future studies. The identification of a consistent global change is an important step toward demonstrating the power of intrinsic parameters to identify subtle differences in cortical morphology. Further region-of-interest studies may be able to address the question as to whether these global changes are driven by specific and consistent regional deficits, or whether the difference identified here represents a pervasive global dysfunction as suggested by functional network analyses (Micheloyannis et al., 2006). In any case, though specific regional differences may emerge in future studies, for our purpose, the demonstration that the consistent group differences are visible at the whole hemisphere level provides clear evidence of the potential value of adopting this method in characterizing morphometry in both healthy and patient populations.

In order to explore and interpret more fully our findings of reduced gyrification in schizophrenia, we contrasted the ratio of intrinsic curvature in the upper and lower layers of the cortex. Although all layers of the cortex contribute to all types of connectivity, there is a non-uniform distribution of connectivity, with upper layers favoring proportionately more short-range connections in comparison to lower layers. Based on the rationale that reduced area indicates less connectivity (less room for connections) (Armstrong et al., 1991), we proposed that a reduced ratio of inner to outer surface area may imply a reduction in short-range connectivity. Our analysis revealed a significantly reduced ratio of pial to white matter intrinsic curvature in the schizophrenic patients (Fig. 4). Under the hypothesis that intrinsic curvature arises from surface expansion, these results may suggest that a relative decrease in the expansion of upper cortical layers, perhaps reflecting a decrease in the proportion of short-range connectivity, is relevant to the pathology of schizophrenia. This result is in keeping with the hypothesis that reduced differential expansion of the cortex reflects a reduction in short-range tangential connectivity (Ronan et al., 2011). Although the exact connection-length distribution has not been quantified in schizophrenia, empirical studies have demonstrated a decrease in presynaptic and dendritic markers, (for discussion see Harrison, 1999), and an overall reduction in neuropil (Buxhoeveden et al., 2000; Selemon and Goldman-Rakic, 1999). In addition, network analyses have also observed longer path-lengths in schizophrenia (Micheloyannis et al., 2006).

Our analysis also gave some insight into the possible forces that govern gyrification. To date the mechanics of gyrification are poorly understood. One theory is that it is driven by cortical expansion, and mediated by axonal tension (Van Essen, 1997): strongly connected regions are pulled together by axons, while less-strongly connected regions “drift”. However a recent study directly measuring axonal tension suggests that axons are not under the required tension to drive folding (Xu et al., 2010). Indeed, the role of axons in gyrification had previously been queried (Barron, 1950) following observations of the developing cortex (of sheep) when isolated in utero from subcortical structures prior to the developmental onset of gyrification. Despite this isolation, essentially normal patterns of gyrification were observed. Thereafter, it was proposed (Richman et al., 1975) that forces internal to the cortex itself drive gyrification, either through differential laminar growth, or inter-neuronal connectivity (Sisodiya, 1995; Toro and Burnod, 2005). In each of these latter models, intra-cortical forces alone drive the formation of gyri and sulci. For this reason, intrinsic curvature is an ideal parameter of gyrification, being solely a function of differential tangential expansion of the cortex. The predictions of these intra-cortical gyrification theories are in keeping with our own observations that intrinsic curvature at the small (millimeter) scale is related to morphology at the centimeter scale. It appears that large-scale cortical features of gyri and sulci are primarily a function of small-scale forces and architecture internal to the cortex, and that these forces themselves may be reflected in the differential geometry of the cortex. We discuss below how this insight leads us to a more detailed interpretation of morphometric abnormalities in our patient groups.

We have previously speculated that intrinsic curvature, as measured here, may relate to the spatial distribution of neurons tangential to the cortical surface and may therefore be a function of the combined effects of neuronal density and differential expansion (Ronan et al., 2011). The findings of the current study are, we feel, interesting and useful irrespective of this theory of what intrinsic curvature may reflect at the neural level. Nonetheless, if our previous speculation (Ronan et al., 2011) is correct, it suggests that the reduced level of intrinsic curvature in schizophrenia reflects altered differential expansion and a correspondingly altered cortical density. This would be in accord with existing neuropathological studies, which have suggested increased cortical density (Selemon et al., 1995) and reduced inter-neuronal separation (Buxhoeveden et al., 2000) in schizophrenia.

In closing, it is important to place these findings, and our suggestion that careful scrutiny of the intrinsic properties of the cortical surface may prove fruitful in developing our understanding of schizophrenia, in the context of the broader literature. The onset of schizophrenia generally occurs between late adolescence and early adulthood. This coincides with a period of synaptic pruning, myelination and arborisation (Blakemore and Choudhury, 2006). Because of the overlap of these connectivity-modulating mechanisms with the onset of clinical symptoms, these neurodevelopmental processes have been linked to the pathophysiology of the disorder. However longitudinal, developmental and epidemiological studies have provided strong evidence that abnormalities exist much earlier — as early as infancy, where developmental milestones are delayed (Jones et al., 1994). Yet, because the classic diagnostic cues are not manifested until much later, the developmental hypothesis of schizophrenia has largely been supposed to be one of early insult followed by a period of latency, with full-blown symptoms emerging later in response to normal, or further abnormal developmental processes specific to adolescence, such as synaptic pruning (Insel, 2010). This picture does not fully address the mechanism whereby cognitive deficits (most resistant to treatment and therefore most likely to be a fundamental hallmark of the disease) exist during a so-called latent (prodromal) period (Khandaker et al., 2011). Rather the evidence suggests that subtle abnormalities present at birth may be slowly augmented throughout development, culminating in severe symptom onset at the apex of development. If this is the case, then small-scale measures of cortical intrinsic curvature may be useful in detecting changes prior to the onset of clinical symptoms. Alternatively by utilizing a measure that has demonstrated consistency and sensitivity, it might be possible to investigate more fully the neurodevelopmental hypothesis of schizophrenia.

## Funding

This work is supported by the Wellcome Trust and the Bernard Wolfe Health Neuroscience Fund, and carried out at the Wellcome/MRC funded Behavioural and Clinical Neurosciences Institute, University of Cambridge. We gratefully acknowledge the assistance of Timothy J. Crow.

## Figures and Tables

**Table 1:** Average values of gyrification (intrinsic curvature skew and *lGI*) broken down by diagnosis for each dataset, with results of linear analysis of group differences with surface area and age as covariates. Text in bold indicates statistically-significant results at alpha = 0.05.

	Adolescents: baseline		Adolescents: follow-up		Adults Group 1		Adults Group 2	
Patients	Controls	Patients	Controls	Patients	Controls	Patients	Controls
Negative curvature skew	− 4.43 ± 0.48	− 4.16 ± 0.51	− 4.71 ± 0.63	− 4.15 ± 0.38	− 5.23 ± 0.69	− 4.8 ± 0.56	− 5.76 ± 0.87	− 4.98 ± 0.29
*(F*_*1,23*_ *= 0.9, p = 0.35)*		*(****F***_***1,23***_ *=* ***8.3****,****p*** *=* ***0.01****)*		***(F***_***1,82***_ ***= 5.5, p = 0.02)***		***(F***_***1,18***_ ***= 7.9, p = 0.01)***	
Positive curvature skew	3.31 ± 0.38	3.14 ± 0.48	3.31 ± 0.45	3.13 ± 0.19	3.64 ± 0.38	3.45 ± 0.31	3.55 ± 0.44	3.46 ± 0.21
*(F*_*1,23*_ *= 1.9, p = 0.18)*		*(F*_*1,23*_ *= 0.03, p = 0.87)*		*(F*_*1,82*_ *= 0.15, p = 0.7)*		*(F*_*1,18*_ *= 0.13, p = 0.72)*	
*lGI*	3.14 ± 0.14	3.25 ± 0.21	3.11 ± 0.16	3.17 ± 0.19	2.94 ± 0.13	3.03 ± 0.2	2.89 ± 0.13	2.85 ± 0.13
*(F*_*1,23*_ *= 2.3, p = 0.14)*		*(F*_*1,23*_ *= 0.001, p = 0.98)*		***(F***_***1,82***_ ***= 4.2, p = 0.04)***		*(F*_*1,18*_ *= 0.21, p = 0.66)*	

**Table 2:** Average values of intrinsic curvature skew broken down by diagnosis and dataset for each of the three levels of down-sampled pial surfaces: For each down-sample level, the average number of vertices per hemisphere and their corresponding area was: down-sample 1: 37,500 vertices of area 2.78 mm^2^; down-sample 2: 9300 vertices of area 9.9 mm^2^; and down-sample 3:2300 vertices of area 31.9 mm^2^.

	Adolescents: follow-up		Adults Group 1		Adults Group 2	
Patients	Controls	Patients	Controls	Patients	Controls
Down-sample 1						
Negative curvature skew	− 3.87 ± 0.48	− 4.17 ± 0.45	− 3.93 ± 0.42	− 3.9 ± 0.36	− 4.48 ± 0.5	− 4.77 ± 0.78
***(F***_***1,23***_ ***= 6.2, p = 0.03)***		*(F*_*1,82*_ *= 0.04, p = 0.84)*		*(F*_*1,18*_ *= 0.04, p = 0.85)*	
Positive curvature skew	3.62 ± 0.7	3.73 ± 0.37	3.74 ± 0.44	3.76 ± 0.6	3.84 ± 0.44	3.49 ± 0.47
*(F*_*1,23*_ *= 2.7, p = 0.12)*		*(F*_*1,82*_ *= 1.9, p = 0.2)*		***(F***_*1,18*_ ***= 6.2, p = 0.02)***	
Down-sample 2						
Negative curvature skew	− 2.72 ± 0.26	− 2.77 ± 0.24	− 2.77 ± 0.22	− 2.79 ± 0.21	− 2.95 ± 0.26	− 3 ± 0.29
*(F = 2.7, p = 0.12)*		*(F = 0.27, p = 0.6)*		*(F*_*1,18*_ *= 0.5, p = 0.5)*	
Positive curvature skew	2.55 ± 0.4	2.41 ± 0.23	2.55 ± 0.33	2.62 ± 0.45	2.5 ± 0.2	2.49 ± 0.22
*(F*_*1,23*_ *= 0.7, p = 0.43)*		*(F*_*1,82*_ *= 2.6, p = 0.1)*		*(F*_*1,18*_ *= 0.02, p = 0.9)*	
Down-sample 3						
Negative curvature skew	− 1.91 ± 0.11	− 1.84 ± 0.19	− 1.87 ± 0.17	− 1.89 ± 0.15	− 1.89 ± 0.16	− 1.93 ± 0.18
*(F*_*1,23*_ *= 2.5, p = 0.13)*		*(F*_*1,82*_ *= 0.4, p = 0.52)*		*(F*_*1,18*_ *= 0.74, p = 0.4)*	
Positive curvature skew	1.38 ± 0.18	1.38 ± 0.28	1.36 ± 0.13	1.37 ± 0.24	1.41 ± 0.15	1.38 ± 0.14
*(F*_*1,23*_ *= 0.01, p = 0.92)*		*(F*_*1,82*_ *= 0.4, p = 0.53)*		*(F*_*1,18*_ *= 0.5, p = 0.49)*	

**Table 3:** Average values of mean curvature skew broken down by diagnosis and each dataset, with results of linear analysis of group differences with surface area and age as covariates.

	Adolescents: follow-up		Adults Group 1		Adults Group 2	
Patients	Controls	Patients	Controls	Patients	Controls
Negative curvature skew	− 1.59 ± 0.11	− 1.55 ± 0.07	− 1.61 ± 0.09	− 1.6 ± 0.11	− 1.56 ± 0.08	− 1.54 ± 0.08
*(F*_*1,23*_*= 8, p = 0.2)*		*(F*_*1,82*_ *= 0.1, p = 0.74)*		*(F*_*1,18*_ *= 0.6, p = 0.4)*	
Positive curvature skew	2.43 ± 0.48	2.3 ± 0.25	2.26 ± 0.38	2.26 ± 0.34	2.42 ± 0.55	2.44 ± 0.73
*(F*_*1,23*_ *= 0.7, p = 0.4)*		*(F*_*1,82*_ *= 0.1, p = 0.8)*		*(F*_*1,18*_ *= 0.03, p = 0.86)*	

## Appendices

### A Intrinsic and mean curvature on the cortical mesh

Both the intrinsic and mean curvatures of the cortical surface were quantified using the Gauss–Bonnet scheme, per vertex on the cortical surface reconstruction. This makes use of the fact that the angles in a triangle on an intrinsically curved surface do not sum to 180°. The greater the degree of intrinsic curvature, the greater the deviation from 180°. Thus the degree of intrinsic curvature per vertex on a cortical surface is defined as the surfeit or deficit of the vertex angle (sum of all triangles which define the vertex) divided by one third the sum of the vertex area (sum of area of triangles which surround the vertex) (Surazhsky et al., 2003) (Eq. (A.1)).

(A.1) $$K_{i}=\frac{2\pi -\sum_{i}\theta_{i}}{\frac{1}{3}\sum_{i}A_{i}}$$

where θ_i_ is the angle subtended by ith vertex, and A_i_ is the area of ith vertex. The corresponding equation for mean curvature per vertex, H_i_ using the Gauss–Bonnet scheme is

(A.2) $$H_{i}=\frac{\frac{1}{4}\sum_{i}\left|\left|e_{i}\right|\right|\beta_{i}}{\frac{1}{3}\sum_{i}A_{i}}$$

where ||e_i_|| is the total length of the vertex edges and β_i_ is the deviation of surface normal (see Surazhsky et al., 2003). In the *FreeSurfer* program the mean and Gaussian curvatures for each vertex are decomposed in to the principle curvatures. Because of per vertex errors which can occur in surface reconstruction, these principle curvatures per vertex are filtered and then multiplied or averaged to give Gaussian and mean curvature per vertex respectively. In this way the effect of single vertex errors is minimized.

In order to filter the principle curvatures a limiting curvature threshold is applied. This threshold is based on the limiting scale at which intrinsic curvature may be reliably calculated, which is defined by an osculating sphere with a radius one-half the diagonal of the unit voxel. In the *FreeSurfer* process, all data are sub-sampled to a unit of side 1 mm giving a limit of resolution of √2/2 mm, equivalent to a radius of curvature of 1.41 mm^− 1^ (Pienaar et al., 2008; Ronan et al., 2011). Although surfaces are reconstructed at a sub-voxel scale (being continuous), this value represents the threshold of reliable curvature values. The principle curvatures are filtered by setting to zero all values which exceed the limiting threshold. After filtering the principle curvature values are then re-multiplied to generate a filtered intrinsic curvature per vertex and a filtered mean curvature per vertex of the cortical reconstruction. Only non-zero intrinsic and mean curvature values are subsequently analyzed.

The reliability of this process was previously investigated. As detailed elsewhere (Ronan et al., 2011), vertex-based measures may be susceptible to artifacts due to local surface quantization effects such as the number of triangles which comprise a vertex. To investigate the sensitivity of intrinsic curvature measures to these effects we applied surface decimation to cortical reconstructions of two species (humans and chimpanzees) to determine whether the distribution of intrinsic curvature skew is robust to changes in how the surface is quantized. The results of our analysis indicated that intrinsic curvature was sensitive to morphological differences between species over several different surface decimation levels.

### B Curvature skew

The use of skew rather than average curvature is prompted by the interaction between the effect of size and the effect of differential expansion on the distribution of curvature values across a surface. To illustrate: consider a brain that is increasing in size. This increase acts to decrease the average intrinsic curvature analogous to the way in which a large sphere will have a lower degree of intrinsic curvature than a small sphere. However because the surface does not expand uniformly (because it is not a uniform structure and undergoes differential growth rates), this means that the degree of differential expansion increases. By definition this also means that the degree of intrinsic curvature increases. Importantly because this change is differential, it implies that the distribution of curvature shapes changes, with proportionately more extreme curvature shapes generated. This change in proportion of curvature shapes favoring more highly-curved shapes acts to increase the average intrinsic curvature. Thus the value of average intrinsic is a combination of the overall brain size increase and surface area expansion which each act in opposite ways on the average intrinsic curvature value.

To avoid this ambiguity we quantify the skew of the intrinsic curvature distribution which reflects only the distribution of curvature shapes and hence can be considered to reflect the degree of differential expansion.

In the cortex the distributions of positive and negative intrinsic curvatures have been demonstrated to be heavily skewed to favor modest degrees of expansion. In other words, by the definition of skew, the median of the distribution is below the average value. As the distribution changes to reflect proportionately more shapes with an increasing degree of intrinsic curvature, there are more values approaching the mean intrinsic curvature and hence the skew is said to decrease (note a distribution has no skew when the median equals the mean). Thus under the hypothesis that intrinsic curvature is a morphological marker of differential expansion, increases in the degree of differential expansion are reflected in less skew of the intrinsic curvature distribution.

These arguments are equally applicable to mean curvature which also has a heavily skewed distribution favoring proportionately fewer sharply folded shapes. Thus mean curvature skew is reported rather than absolute values of mean curvature.
